# Upregulation of G Protein-Coupled Estrogen Receptor by Chrysin-Nanoparticles Inhibits Tumor Proliferation and Metastasis in Triple Negative Breast Cancer Xenograft Model

**DOI:** 10.3389/fendo.2020.560605

**Published:** 2020-09-15

**Authors:** Kyoung Mee Kim, Joohee Jung

**Affiliations:** College of Pharmacy, Duksung Women's University, Seoul, South Korea

**Keywords:** chrysin-nanoparticle, triple-negative breast cancer, metastasis, tumor progression, G protein-coupled estrogen receptor

## Abstract

Triple-negative breast cancer (TNBC) is associated with a high mortality rate among women globally. TNBC shows a high rate of recurrence and distant metastasis. Particularly, the chemotherapy is limited because hormone therapy of breast cancer is ineffective. Thus, an effective chemotherapeutic agent is needed for tumor suppression. Chrysin-nanoparticles (chrysin-NPs) were investigated for their inhibitory effect on a MDA-MB-231-derived xenograft model. To gain insight into the underlying mechanisms, we conducted human matrix metalloproteinase (MMP) array, western blot, and immunohistochemistry analysis. Furthermore, *in vivo* imaging was used to monitor the chemotherapeutic efficacy of chrysin-NPs in a metastasis mouse model. Chrysin-NPs significantly inhibited the proliferation of MDA-MB-231 cells *via* the PI3K/JNK pathway and induced cell death through the p53-apoptosis pathway, leading to delayed MDA-MB-231-derived tumor growth. Interestingly, chrysin-NPs significantly induced G protein-coupled estrogen receptor (GPER) expression, which suppresses MMPs and NF-κB expression. Chrysin-NPs acted as effective metastasis inhibitors. Our results suggest that chrysin-NPs may be used as an effective adjuvant formulation to inhibit TNBC progression.

## Introduction

Breast cancer is a leading cause of deaths in woman worldwide (1, 2). Breast cancer is divided into four different subtypes; luminal A (estrogen receptor (ER) and progesterone receptor (PR) positive, human epidermal receptor 2 (HER2) negative and low Ki-67 level), luminal B (ER and PR positive, HER2 positive or negative and high Ki-67 level), HER2 enrich (ER and PR negative), and triple negative (ER, PR, and HER2 negative) (3, 4). The therapeutic modalities for triple-negative breast cancer (TNBC) are limited to surgery or conventional chemotherapy as TNBC patients will not respond to endocrine therapy or receptor targeting treatments (5). TNBC accounts for 15% of all cases of breast carcinoma (6), has the poorest overall survival of all breast cancer subtypes (7), and has the highest rates of epithelial-to-mesenchymal transition (EMT) metastasis, possibly resulting from the remarkable phenotypic similarity between TNBC cells and mammary stem cells (8).

Therefore, development of targeted therapies for TNBC is urgently needed. The course of tumor metastasis entails a series of stages that lead to the formation of secondary tumors in distant organs (9). The process of invasion is instigated as the original tumor cells pass through the basement membrane and extracellular matrix, journey through the circulatory system, and attach at a new location to proliferate and produce secondary tumors (10, 11). Resultantly, research efforts focused on identifying and understanding the mechanisms concerned in tumor cell invasion may lead to the development of novel approaches to inhibit tumor progression in TNBC patients. The key enzymes responsible for ECM breakdown are matrix metalloproteinases (MMPs), a family of zinc- and calcium-dependent endopeptidases involved in the regulation of cell growth, migration, angiogenesis and invasion (11–13). The role of MMPs in a variety of cancers has been reviewed elsewhere (14, 15). In breast cancer, the expression levels of MMPs were reported to be higher than in normal breast tissues (16, 17). For instance, MMP-1,−2,−7,−9,−10,−11,−13,−14, and−15 were documented for their contribution to breast cancer proliferation and metastasis (18–21). Previous studies have reported that the G protein-coupled estrogen receptor-1 (GPER, formerly known as GPR30) was associated with disease progression in cancer patients (22). A wide number of natural and synthetic compounds, including estrogens and anti-estrogens, elicit stimulatory effects in breast cancer through GPER upregulation and activation (23). Estrogen signaling and ERα are well-documented for their contribution to the progression of ER-positive breast cancers (24). In TNBC patients who lack the expression of ER, stimulation by estrogen or/and anti-estrogen is mediated via GPER (24–26), supporting the contributory role of GPER in TNBC disease progression (26). Other authors have also reported that GPER is involved in the development and/or proliferation of renal (27), endometrial (28, 29), and ovarian cancers (30, 31).

Chrysin, also called 5,7-dihydroxyflavone, is a flavone found in the clock flower and in honeycomb (32). It has various physiological activities including anti-inflammatory, antioxidant, hypoglycemic and anti-aromatase activity (32). The compound was reported to inhibit the proliferation of non-small cell lung cancer cells (33, 34). In another study, it was confirmed that invasion and migration of TNBC MDA-MB-231 cells were reduced in the presence of low concentrations of chrysin (7). However, chrysin is difficult to apply to *in vivo* system because it is insoluble in water. Thus, chrysin is effective, but its reports are limited (35, 36). In order to overcome the disadvantages (solubility and degradation), many researchers are studying to improve the efficacy and effectiveness of the drug by using a drug delivery system (37, 38). Among many drug delivery systems, polymers that synthesize biodegradable polyesters have been applied for many years (39–41), nanoparticles composed of hydrophobic poly (ε-caprolactone) (PCL) and hydrophilic poly(ethylene glycol) (PEG) among several polymers (42, 43) have been reported to be good potential carriers for anti-cancer agents (39, 44). PCL is a biodegradable, biocompatible, hydrophobic and non-toxic thermoplastic polyester (45). PEG is a common constituent for the hydrophilic outer shell and is known to reduce the adhesion of plasma proteins, solubility in water and organic solvents, stabilization of particles, and lack of toxicity (46, 47). In addition, it has been reported that polymeric nanoparticles with hydrophilic PEG outer shell can increase the circulation time of the hydrophobic anticancer agents in the body and prevent recognition by macrophages of the reticuloendothelial system (RES) after intravenous administration (47, 48). Additionally, PCL-PEG nanoparticles (without drug) were non-toxic in the liver and kidney of mice (49). Several researches reported that polymer could improve the bioavailability of chrysin (33, 50).

The aim of our study was to determine whether chrysin-nanoparticles (chrysin-NPs) may be used as an effective adjuvant formulation to inhibit the progression and metastasis of TNBC using a xenograft model.

## Materials and Methods

### Preparation of Chrysin-NPs

**Figure 1:**
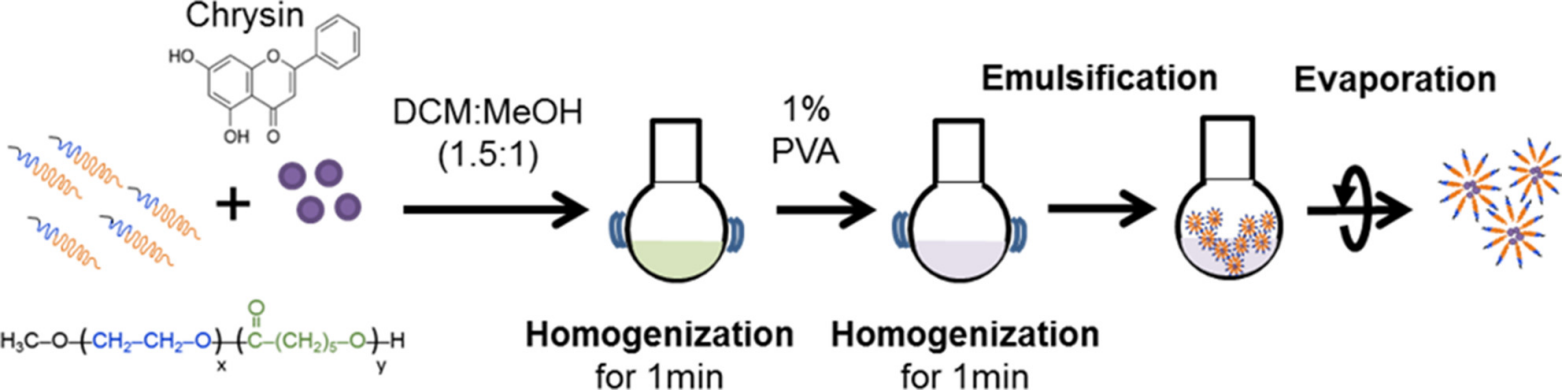
Production flowchart of chrysin-loaded nanoparticles.

Chrysin-NPs were produced as previously described, with minor modifications (Figure 1) (33). In brief, chrysin and polyethylene glycol-β-polycaprolactone copolymer (mPEG-PCL, PolySciTech, West Lafayette, IN, USA) were mixed at a ratio 1: 10 (w/w) in dichloromethane (DCM): methanol (1.5:1 v/v, Duksan reagent, Gyeonggi-do, Korea) solution. The mixture was then homogenized for 1 min and a solution of 1% polyvinyl alcohol was added to create an emulsion. Chrysin-NPs micelles were obtained upon evaporation. Chrysin-loading efficiencies were calculated as a previous report (33). Chrysin-NPs were prepared when it used. The average of encapsulated efficiency was 61.1% in chrysin-NPs.

### Cell Culture

MDA-MB-231 and MDA-MB-231_luc cells (luciferase-expressing cells, kindly provided from Prof. Moon, Duksung Women's University) were maintained in RPMI-1640 medium (GenDEPOT, Barker, TX, USA) containing 10% fetal bovine serum (YOUNGINFRONTIER, Seoul, Korea) and 1% penicillin/streptomycin (GenDEPOT) in a 5% CO_2_ humidified atmosphere at 37°C.

### MTT Assay

MDA-MB-231 cells (5,000 cells/well) were seeded in 96-well plates and incubated for 24 h. Chrysin (Sigma-Aldrich, St Louis, MO, USA), NP or chrysin-NPs were then added for a 48 h-incubation period. G-1 and G-15 (Cayman Chemical, Michigan, USA) were added for a 24 and 48 h-incubation period. MTT (Sigma-Aldrich) was added to the media for a further 3 h, and the supernatant was gently removed and discarded. DMSO (Sigma-Aldrich) was added and absorbance (560 nm) was determined using a microplate reader (Infinite M200 PRO; Tecan Inc., Grödig, Austria). The data were represented a mean ± standard deviation (*SD, n* = 4).

### Experimental *in vivo* Study

All animal experiments were approved by the Institutional Animal Care and Use Committee of Duksung Women's University in accordance with the guidelines for the care and use of laboratory animals. Five-weeks-old female Balb/c nude mice were obtained from JUNGAH BIO (Gyeonggi, Korea). Healthy mice were left to acclimatize for 1 week prior to any procedural work. The conditions in the laboratory were 20°C, 50% humidity, and a 12/12-h light/dark cycle. Diet was provided with drinking water *ad-libitum*.

### *In vivo* Tumor Growth Monitoring

MDA-MB-231 cells (5 × 10^6^ cells/mouse) were orthotopically implanted into the mammary fat pads of mice. When MDA-MB-231-derived tumor reached at volume of 150–200 mm^3^, mice were randomly divided into 2 groups (*n* = 6/group). Tumor sizes were measured three times per week. Tumor volumes were calculated using the following equation:

Tumor volume (mm^3^) = (Length × Width^2^) × 0.5

The data were represented as mean ± standard deviation (*SD*).

### Luminescence Measurement Using *in vivo* Imaging

MDA-MB-231_luc cells (1 × 10^5^ cells/mice) were intravenously injected into the tail vein of NGRA mice (5-weeks-old, female). To track tumor cell movement, 100 μL of D-luciferin (XenoLightTM D-luciferin potassium salt, PerkinElmer, EU) was intraperitoneally injected two times per week to NGRA mice bearing MDA-MB-231_luc cells. Mice were anesthetized with isoflurane (TerrellTM, Piramal, PA, USA) and their luminescence was quantified within 10 min of the injection using an *in vivo* imaging system (VISQUETM *in vivo* Elite, Vieworks, Gyeonggi-do, Korea). When luminescence was detected, mice were randomly divided into two groups (*n* = 3/group).

### Administration of Chrysin-NPs

The mouse control group was injected with saline and the treated group was intravenously injected with chrysin-NPs (10 mg/kg) three times per week for 20 days. Mouse body weights were recorded three times per week.

### Western Blot Analysis

Tumor tissues were homogenized in a radioimmunoprecipitation assay (RIPA) buffer (GenDEPOT) containing a protease and phosphatase inhibitor cocktail (GenDEPOT). The extracted proteins were quantified using a bicinchoninic acid (BCA) assay (Thermo Scientific, Waltham, MA, USA). Proteins were then separated using 12% SDS-polyacrylamide gel electrophoresis (SDS-PAGE) and transferred to polyvinylidene fluoride (PVDF) membranes (Millipore, Darmstadt, Germany). Membranes were blocked with 5% skimmed milk in tris-buffered saline supplemented with polysorbate 20 (TBST) (50 mM Tris-HCl pH 7.4, 150 mM NaCl, 0.1% Tween20), washed and incubated with anti-p-JNK antibody (sc-12882, Santa Cruz Biotechnology, Texas, USA, 1:1,000), anti-JNK antibody (sc-571, Santa Cruz Biotechnology, 1:1,000), anti-Akt antibody (H-136, sc-8312, Santa Cruz Biotechnology, 1:1000), anti-p-Akt (C400, Cell signaling technology, MA, USA, 1:1000), anti-GSK-3α/β antibody (sc-7291, Santa Cruz Biotechnology, 1:1000), anti-NK-κB antibody (MAB3026, Millipore, Darmstadt, Germany, 1:1000), anti-MMP-10 antibody (sc-80197, Santa Cruz Biotechnology, 1:1,000), anti-MMP-2 antibody (MAB13405, Millipore, 1:1000), anti-PI3K antibody (C73F8, Cell signaling technology, 1:1000), anti-GPER antibody (ab188999, Abcam, USA, 1:1000) or anti-β-actin antibody (A4331, Sigma Aldrich, 1:5,000) at 4°C, overnight. The membrane was incubated with a secondary antibody (1:3,000) at room temperature for 3 h. Blots were visualized using enhanced chemiluminescent (ECL) solution and observed using the ChemiDocTM imager (FluorChemE, Germany). Images of western blots were quantified using Image J software.

### Human Mmatrix Metalloproteinase Antibody Array

MMP-related proteins were detected using a human MMP array kit (RayBiotech, USA). The extracted tumor tissues were lysed using a RIPA buffer (GenDEPOT) containing a protease and phosphatase inhibitor cocktail (GenDEPOT). The resulting lysate was applied to the membrane array kit and incubated overnight at 4°C. After several washings, the membrane was incubated with HRP-Streptavidin for 2 h at room temperature, washed and further incubated with the kit detection buffer for 2 min at room temperature. MMP-related protein expression was observed using the ChemiDocTM imager.

### Tissue Preparation

Tumor tissues were isolated and embedded into optical cutting temperature (OCT) compound (Leica, Nussloch, Germany) or paraffin. Frozen or paraffin blocks were sectioned into 5 μm slices.

### TUNEL Assay

TUNEL assay was performed as previously described (33). Sections were hydrated with 100, 90, and 70% ethanol, bathed in 3% H_2_O_2_/distilled water (DW), washed with DW, and then incubated in DW at 60°C for 1 h. The sections were subsequently cooled to room temperature for 1 h, treated with terminal deoxynucleotidyl transferase (TdT) labeling buffer, and then incubated in TdT (Sigma)/biotinylated deoxyuridine (Roche Diagnostics, Mannheim, Germany) for 1 h at 37°C in a humidified chamber. The reaction was stopped using terminating buffer, and slides were washed with DW. The tissue sections were then blocked with 2% bovine serum albumin (BSA; bioWORLD, Dublin, OH, USA) in PBS, washed and incubated with the ABC complex also diluted in PBS. A final wash was carried out using 0.05 M Tris buffer, and color development with DAB substrate (Vector laboratories, Inc., Burlingame, CA, USA). Results were examined under a microscope (Leica). Images of tissues (*n* = 5, each group) were quantified using Image J software and plotted as percent of stained area.

### Immunohistochemistry

Immunohistochemical staining was performed as previously described (33). Tissue sections were incubated with anti-Ki-67 antibody (ab16667, Abcam, 1:100) and the expression of Ki-67 was visualized using a DAB peroxidase substrate kit (Vector laboratories). Cell nuclei were visualized using hematoxylin-based counterstain solution (Sigma-Aldrich).

### Statistical Analysis

All data were analyzed using Prism7 (GraphPad Software Inc., San Diego, CA, USA) using Student's *t*-test or ANOVA test. Differences were considered statistically significant when *p*-value was inferior to 0.05.

## Results

### Chrysin-NPs Inhibit Metastasis-Related Signaling and Induce Cell Death in MDA-MB-231 Cells

**Figure 2:**
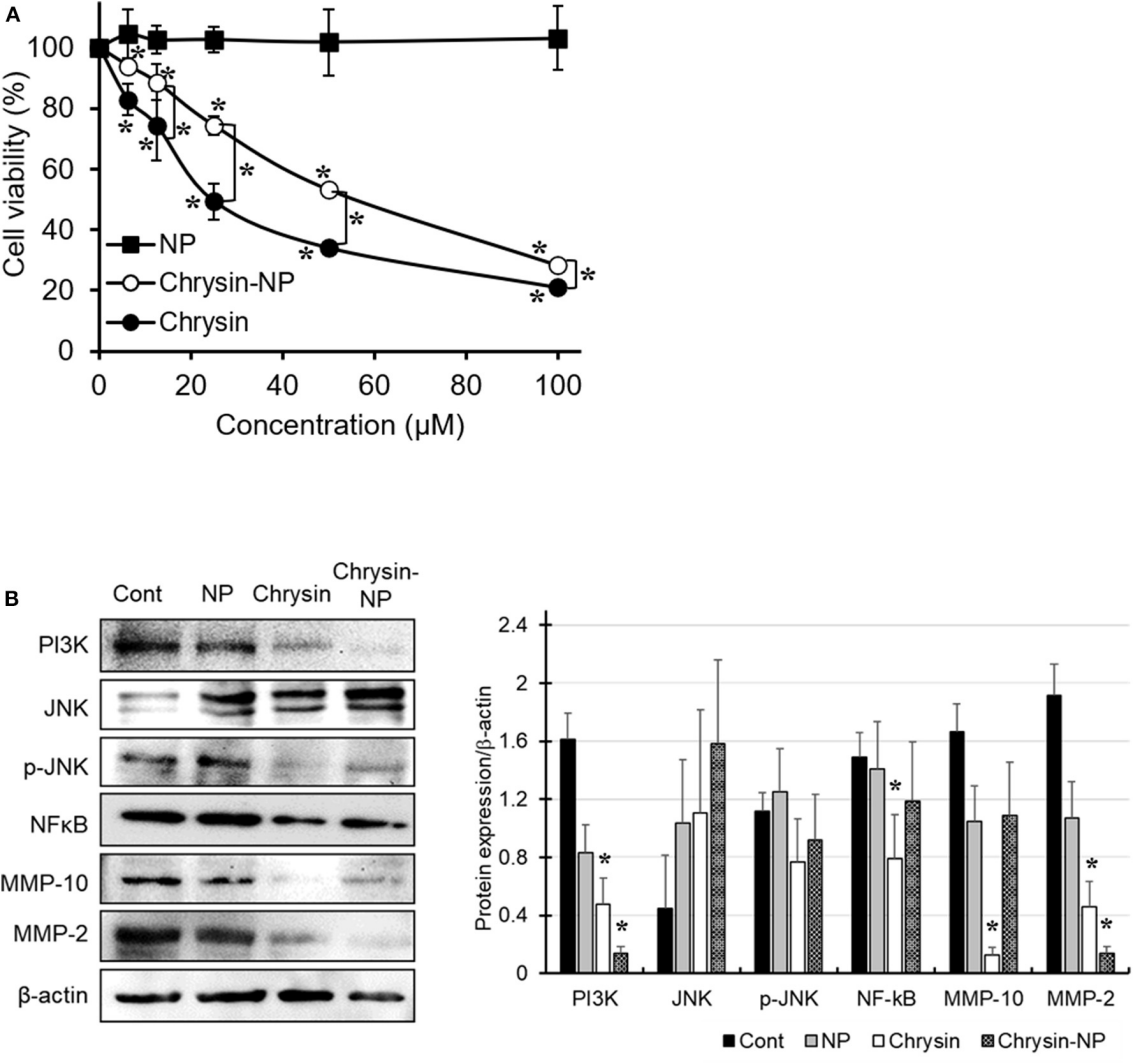
Chrysin-NPs inhibit cell viability and PI3K/JNK signaling pathway. MDA-MB-231 cells were treated with chrysin or chrysin-NPs for 48 h. **(A)** Cell viability was assessed by MTT assay. Data were represented as mean ± S.D. (*n* = 6). ^*^p < 0.05 (ANOVA). **(B)** The expression levels of PI3K/JNK signaling proteins were detected by western blot analysis. Data were represented as mean ± S.D. (*n* = 3). ^*^p < 0.05 (ANOVA).

The anti-cancer properties of chrysin-NPs were investigated in MDA-MB-231 TNBC-like cells using an MTT assay (Figure 2A). Our results showed that chrysin-NPs inhibited cell viability in a dose-dependent manner, in a similar manner to chrysin itself. No change in cell viability was detected in the NP-vehicle control wells. These observations suggest that, when delivered in a nanoparticulate format, chrysin maintained its anti-cancer properties *in vitro*. Upon western blot analysis of the expression levels of molecules known to be involved in cancer progression (Figure 2B), we found that PI3K and NF-κB levels were found to be lower upon treatment with chrysin-NPs. Similarly, the expression levels of MMP-10 and MMP-2, which are known to play a role in invasion and metastasis, were also in decline.

### Chrysin-NPs Delay Tumor Growth Through Apoptosis

**Figure 3:**
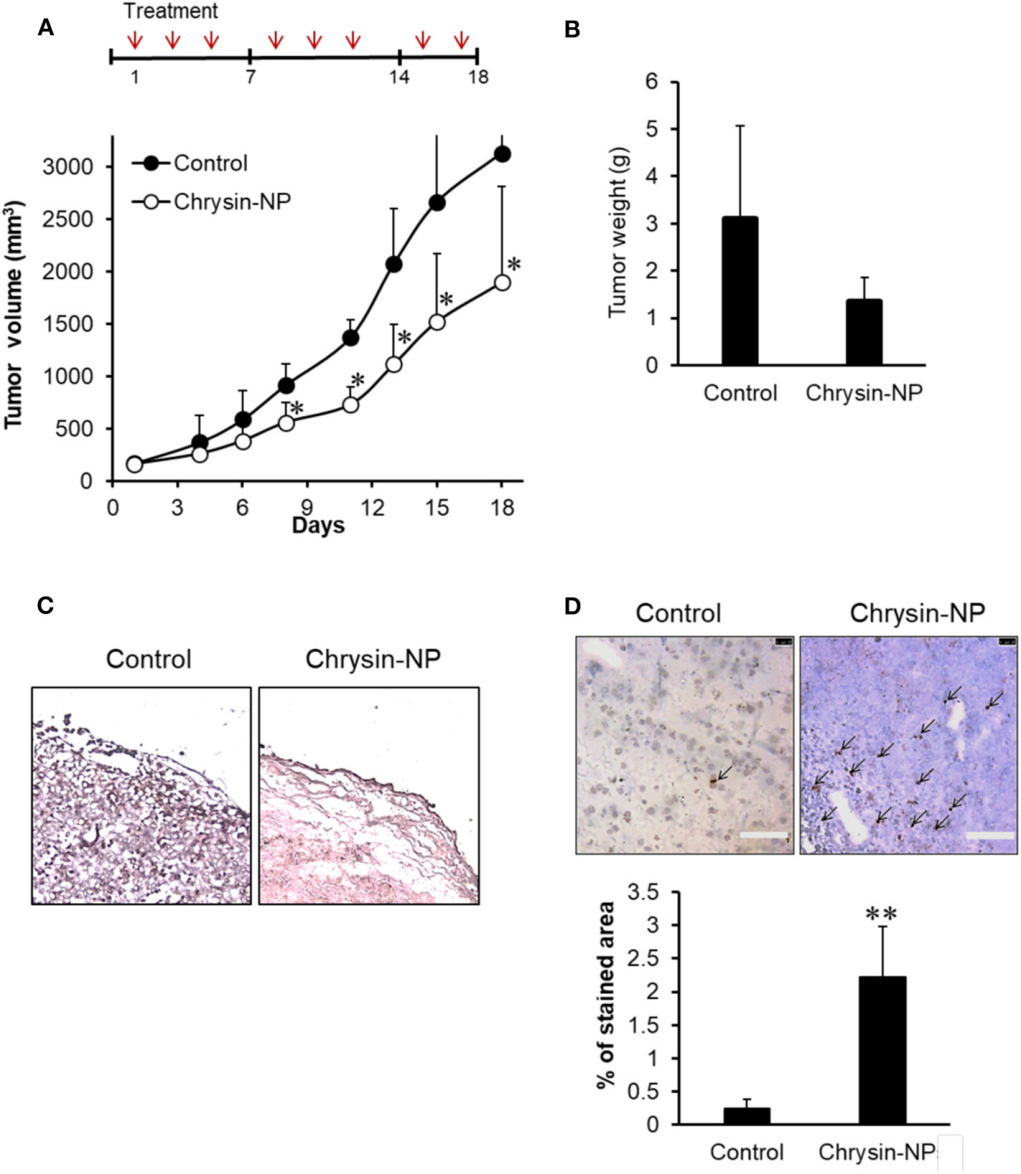
Chrysin-NPs suppress tumor growth in MDA-MB-231-derived xenograft models. **(A)** Administration schedule of chrysin-NPs in mice. Chrysin-NPs (10 mg/kg) were administrated intravenously every other day, three times per week for 18 days. Growth curve of tumors in mice treated with chrysin-NPs. Data are represented as mean ± S.D. (*n* = 6). ^*^p < 0.05 (Student's *t*-test). **(B)** Comparison of tumor weights between sham-treated control and chrysin-NP treated group. Data are represented as mean ± S.D. (*n* = 4). **(C)** Expression of Ki-67 in MDA-MB-231-derived tumor tissues visualized by immunohistochemistry. Scale bar, 100 μm. **(D)** The brown spots (black arrows) denotes TUNEL-positive apoptotic cells. Scale bar, 100 μm. Apoptotic cells are quantified. Data are represented as mean ± S.D. (*n* = 5). ^**^p < 0.005 (Student's *t*-test).

To evaluate the chemotherapeutic efficacy of chrysin-NPs, tumor growth was compared between control and chrysin-NP-treated mice. A chrysin only treated group was omitted in this study on the basis that in a previous study, chrysin-NPs were shown to be injectable and effective at preserving its biological activities *in vivo* (33). Upon repeated administration of chrysin-NPs, a significant growth delay was observed in MDA-MB-231-derived tumors (Figure 3A). Additionally, a decrease in tumor weight was detected in mice treated with chrysin-NPs, but the data were not significantly different due to the large difference in individual data (Figure 3B). Similarly, the expression level of Ki-67, a proliferation marker, declined in mice that were administered with chrysin -NPs compared to control mice (Figure 3C). The tumor tissues treated with chrysin-NPs presented the morphological hallmarks of apoptosis (Figure 3D, black arrow). Apoptotic cells (stained area) were quantified (Figure 3D, graph). The tumor tissues treated with chrysin-NPs significantly increased apoptosis. Altogether, our results indicate that chrysin-NPs significantly repressed TNBC-derived tumor growth through inhibition of proliferation and induction of apoptosis.

### Chrysin-NPs Suppress Metastatic Signaling in MDA-MB-231-Derived Tumors

**Figure 4:**
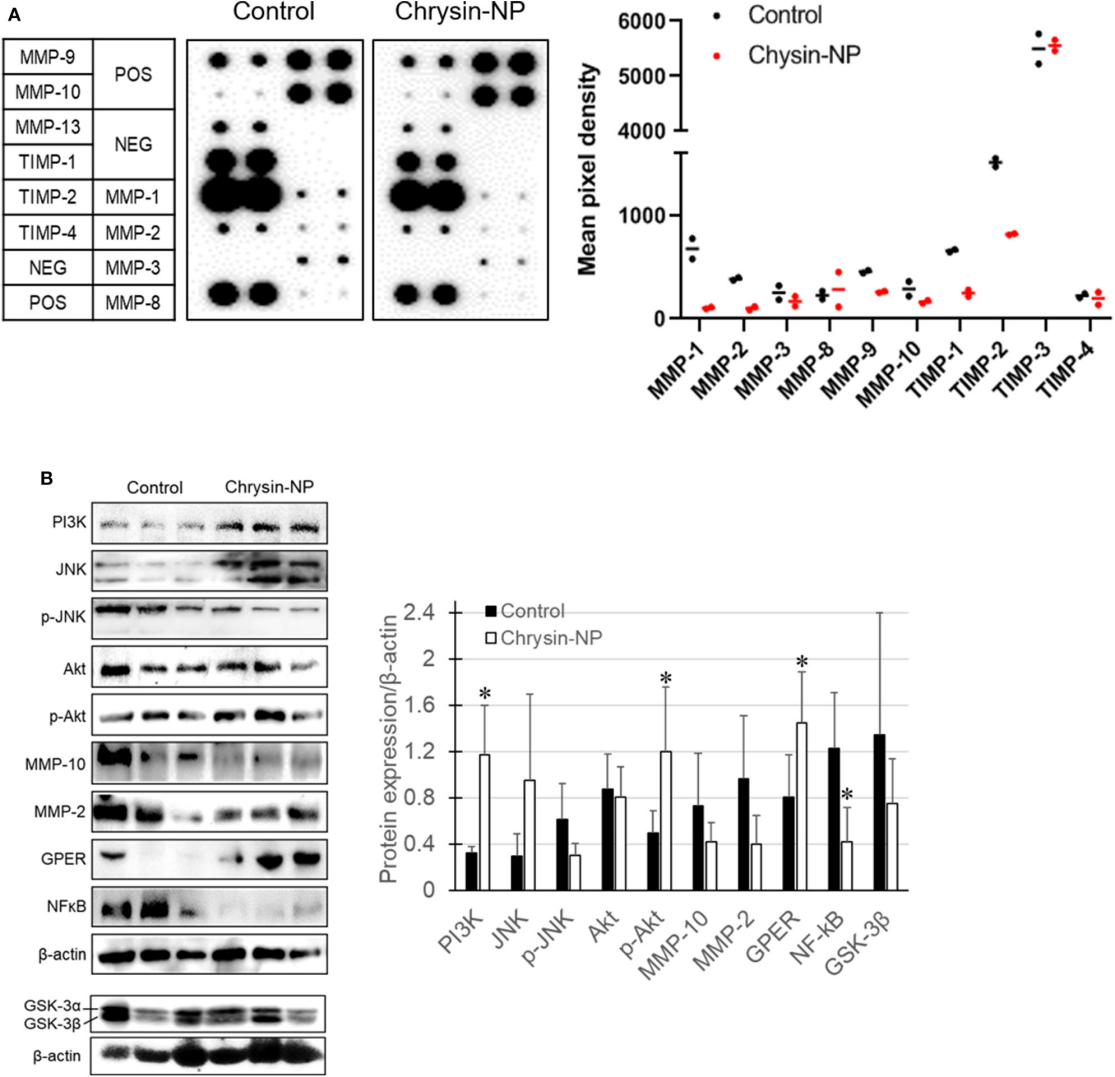
Chrysin-NPs inhibit metastatic signaling pathways *via* GPER. **(A)** Map of MMP and TIMP arrays and their respective expression level. Relative expression of MMPs and TIMPs upon treatment with chrysin-NPs. **(B)** Metastasis-related protein levels upon incubation with chrysin-NPs. The quantification was performed through the Imaga J (■, control group; □, chrysin-NP group, left panel). Relative expression level of proteins are represented as mean ± S.D. (*n* = 6). ^*^p < 0.05 (Student's *t*-test).

We previously showed (Figure 2) that chrysin-NPs exerted inhibitory effects on cancer progression *in vitro*. Here we sought to determine whether chrysin-NPs could exert any inhibition on the invasion mechanisms *in vivo*. The expression levels of MMPs were measured to evaluate invasion and migration in MDA-MB-231-derived tumor tissues. As shown in Figure 4A, the expression levels of MMP-1, 2, 3, 9, 10, and 13 declined in the tumor tissues isolated from mice treated with chrysin-NPs when compared with the control mice. On the other hand, the expression levels of MMP-8 (neutrophil collagenase) were on the rise upon treatment with chrysin-NPs, while expression of tissue inhibitor of metalloprotease (TIMP)-1 decreased. To gain insight into the mechanism of action of chrysin-NPs, we investigated the expression levels of markers of the PI3K/Akt pathway and GPER pathway on the basis of their reported contribution to MMPs expression, invasion, migration and metastasis (Figure 4B). Our results indicated a rise in the expression of PI3K and phospho-Akt (p-Akt), and a decrease in the expression of GSK-3β and NF-κB in tumors collected from mice treated with chrysin-NPs.

### Chrysin-NPs Inhibit Metastasis

**Figure 5:**
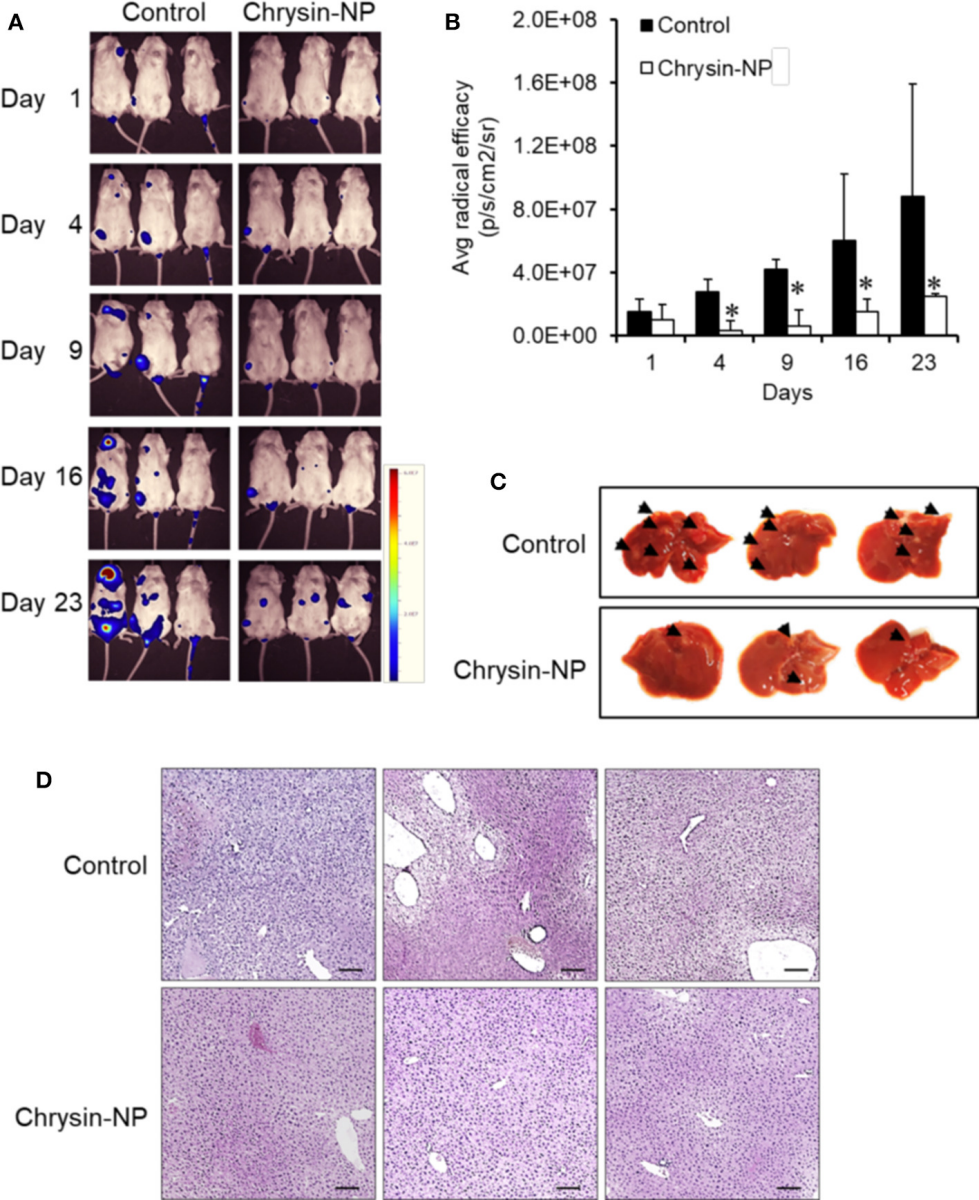
Chrysin-NPs suppress TNBC-related metastasis. **(A)** *In vivo* imaging of luminescence in mice administered with MDA-MB-231_luc cells. **(B)** Quantitation of luminescence (*n* = 3/group). Data are represented as mean ± S.D. ^*^P < 0.05 (Student's *t*-test). **(C)** Photograph of liver **(D)** Histological analysis of liver tissues stained with H& E. Scale bar, 100 μm.

We next sought to investigate the effect of chrysin-NPs on TNBC-derived metastasis *in vivo*. Chrysin-NPs treated mice were observed using an *in vivo* imaging system. While control mice showed a time-dependent increase in luminescence hence metastasis, the mice treated with chrysin-NPs showed a significant lower incidence of metastasis (Figures 5A,B). In particular, the number and density of metastatic spots (Figure 5C, black arrows) observed in the liver tissues of control mice were higher than those observed in the liver tissues of chrysin-NPs-treated mice. The liver tissues of control mice showed tumor nests and loose blood vessels, while the liver tissues of chrysin-NPs treated mice didn't (Figure 5D).

## Discussion

**Figure 6:**
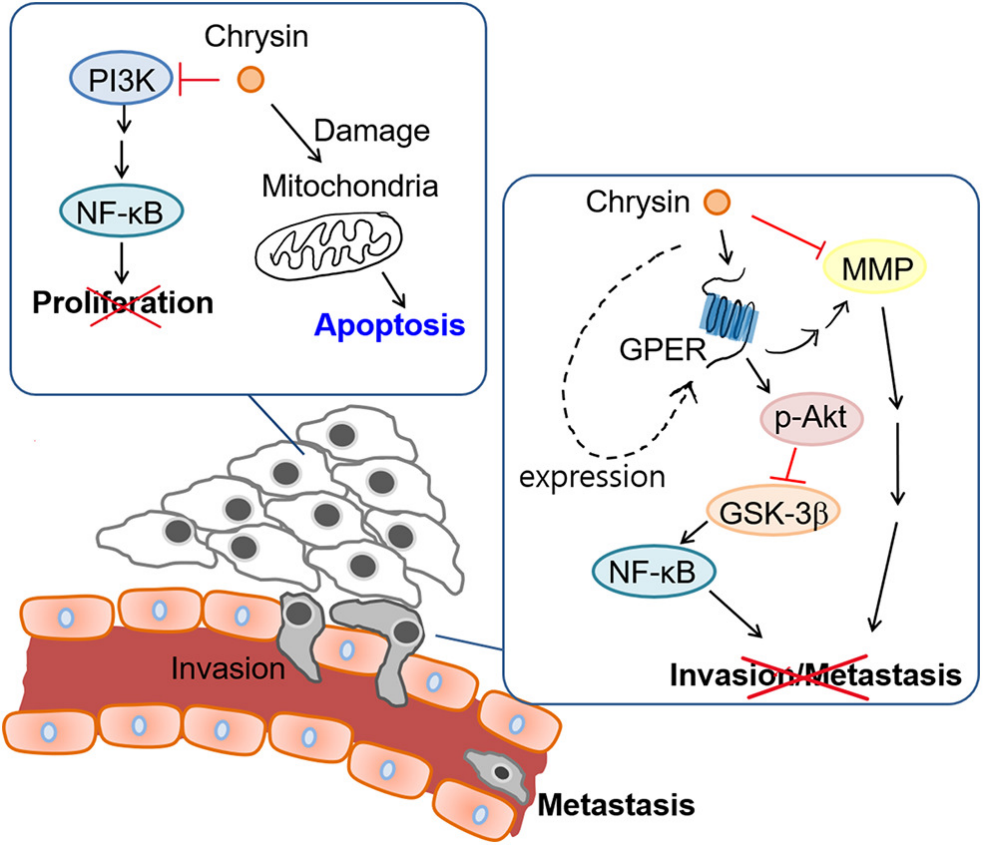
Proposed model for the mechanism of tumor suppression by Chrysin-NPs in TNBC cells.

The aim of our study was to investigate the anti-cancer properties of chrysin encapsulated into mPEG-PCL nanoparticles. Our results showed that chrysin-NPs suppressed TNBC progression via activation of the GPER signaling pathway *in vivo*. Chrysin-NPs induced apoptosis in MDA-MB-231-derived tumors (Figure 3D) and inhibited tumor growth in a xenograft model (Figure 3A). Furthermore, our data indicate that chrysin-NPs suppressed metastasis (Figure 5). Using these results, the proposed model was described for the inhibitory mechanism of tumor progression by chrysin-NPs in xenograft model (Figure 6).

In a previous study, we found that both chrysin-NPs and chrysin could delay tumor growth in a lung cancer xenograft model (33). Here, we showed that chrysin-NPs could inhibit the early stages of TNBC disease progression. Furthermore, chrysin-NPs lowered the expression levels of MMPs (Figure 4A). Several studies have reported that MMPs played an important role in cancer proliferation, angiogenesis, and metastasis in various cancers. Here chrysin-NPs inhibited the expression of MMP-1,−2,−3,−9,−10, and−13, while the expression of MMP-8 was higher in our MDA-MB-231-derived xenograft model than in the control group (Figure 4A). In particular, the expression levels of MMP-2 and−10 were consistently lower upon treatment with chrysin-NPs both *in vitro* and *in vivo* (Figures 2, 4). These results are in line with previous studies (7). Down regulation of MMP-1,−2,−3,−9,−10, and−13 was associated with cancer progression and poor prognosis in breast cancer patients (16, 17, 51, 52). On the other hand, MMP-8 was shown to exert anti-proliferative and inhibitory activities on the spread of cancer cells to tissues, with a net inhibitory effect on metastasis (53). TIMP-1 was described for its inhibitory activity on metalloproteinase, but there have been conflicting reports on its anti-apoptotic activity and its role in stimulating cell proliferation in breast cancer (10, 54). In the present study, the decreased expression of TIMP-1 upon treatment with chrysin-NPs was consistent with its anti-apoptotic activities. These results reinforce our observation that chrysin-NPs exert multiple suppressive effects on cancer progression *in vivo* through inhibition of TIMP-1, cell proliferation, and metastasis via downregulation of MMPs.

We next sought to elucidate the molecular mechanisms involved in MMPs signaling upon chrysin-NP treatment. The JNK signaling pathway is well-documented for its role in cancer progression and development. Activation of the JNK pathway was confirmed by western blot analysis of tumor tissue homogenates: expression of total-JNK was not significant change upon treatment with chrysin-NPs while the expression of p-JNK was lower. Furthermore, expression levels of MMP-2, MMP-10, and NF-kB were lower in the chrysin-NPs-treated group as compared with the control group. Previous authors have reported that MMP-2 was regulated *via* PI3K and NF-kB pathway in breast cancer (55) and the inhibition of MMP-9 was associated with the inhibition of p-JNK (56). Our results also showed that the reduction of PI3K, p-JNK, and NF-kB by chrysin occurred the inhibition of MMPs in MDA-MB-231 cells. However, chrysin-NPs induced PI3K/p-Akt expression level in MDA-MB-231-derived tumor tissues (Figure 4B). The result might be to cell-cell interaction, and crosstalk of cancer cell signaling and cells of tumor microenvironment (immune cells, fibroblast, stem cells). Chrysin-driven inhibition of MMP-10 was reported to be associated with the inhibition of p-Akt in breast cell lines (7). Our results also highlighted that chrysin-NPs induced higher *in vivo* activation of the PI3K/Akt signaling pathway compared to chrysin alone (Figure 4B). Altogether, our results concur with the observation that chrysin-NPs may be used as an effective suppressor of cancer progression.

Next, we investigated the anti-metastatic efficacy of chrysin-NPs *in vivo*. As shown in Figure 5, the administration of chrysin-NPs led to significant inhibition of tumor cell metastasis. Downregulation of MMPs and TIMP-1 expressions upon treatment with chrysin-NPs was associated with lower levels of NF-kB. We also investigated the changes in expression of GPER upon treatment with chrysin-NPs. GPER is an alternate estrogen receptor with a structure distinct from the two canonical estrogen receptors, ERα and ERβ, and present multiple functions in a variety of tissues. Estrogen and/or anti-estrogen-induced effects mediated via GPER have been previously reported in TNBC. GPER is also known to regulate estrogen signaling during the progression of TNBC, but the exact mechanisms remain unclear. Previous authors have reported that GPER activation by G-1 resulted in inhibition of metastasis and EMT via NF-κB (57), and suppression of tumor proliferation in breast cancer (29, 58, 59). GPER activation is also known to inhibit metastasis and proliferation in endometrial, ovarian, liver and adrenocortical cancers (28, 31, 60, 61). The activation of GPER, another target receptor in TNBC, was shown to inhibit cell migration and invasion (22). In contrast, other authors have reported that GPER activation could induce invasion and migration in kidney cancer, with a poor prognosis for the patients (24). Here, we showed that the administration of chrysin-NPs led to higher GPER expression levels in tumor tissues (Figure 4B), and lower expression levels of NF-κB, a signaling molecule downstream of GPER. As shown in Figure 6 and Supplementary Data, PI3K/Akt signaling activated by GPER inhibited GSK-3β, an inhibitor of NK-kB (57, 62). The result suggests that chrysin could induce the GPER expression and suppress the metastasis of TNBC cells as a GPER agonist.

Our results showed that the loading of chrysin onto mPEG-PCL nanoparticles resulted in the enhancement of its anti-cancer properties. Most importantly, we found that chrysin-NPs activated a GPER-mediated NF-κB signaling pathway. These results strongly support that chrysin-NPs exert inhibitory effect on tumor growth and prevent metastasis. Hence our study supports the use of chrysin-NPs as a novel chemo-adjuvant for the treatment of TNBC patients.

## Data Availability Statement

All datasets generated for this study are included in the article/Supplementary Material.

## Ethics Statement

The animal study was reviewed and approved by the Institutional Animal Care and Use Committee of Duksung Women's University.

## Author Contributions

JJ: conceptualization, formal analysis, data curation, writing review, editing, visualization, supervision, project administration, and funding acquisition. KK: methodology, validation, and investigation. KK and JJ: writing original draft preparation. All authors have read and agreed to the published version of the manuscript.

## Conflict of Interest

The authors declare that the research was conducted in the absence of any commercial or financial relationships that could be construed as a potential conflict of interest.
