# Supplementary material for: Upregulation of G Protein-Coupled Estrogen Receptor by Chrysin-Nanoparticles Inhibits Tumor Proliferation and Metastasis in Triple Negative Breast Cancer Xenograft Model
Source: Front Endocrinol (Lausanne). 2020 Sep 15;11:560605. doi: 10.3389/fendo.2020.560605 (PMC7522162; doi:10.3389/fendo.2020.560605)
Supplement: Supplementary file 1 [file Data_Sheet_1.PDF]

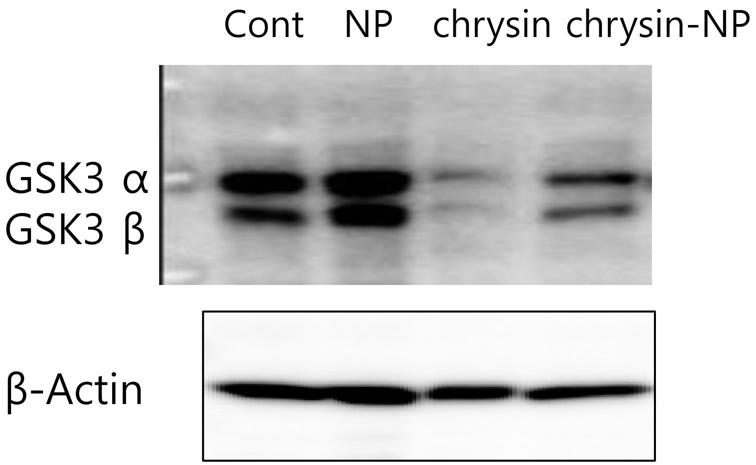

Supplementary Figure 1. Decline of GSK-3b expression by chrysin-NP in MDA-MB-231 cells. The condition of samples is equal to that in Figure 2.
